# Maternal infections in pregnancy and the risk of sudden unexpected infant death in the offspring in the U.S., 2011–2015

**DOI:** 10.1371/journal.pone.0284614

**Published:** 2023-04-21

**Authors:** Maggie Weatherly, Anusua Trivedi, Ratna Chembrolu, Sanjana Gupta, Jan-Marino Ramirez, Juan M. Lavista Ferres, Tatiana M. Anderson, Edwin A. Mitchell

**Affiliations:** 1 Master of Science in Data Science, University of Washington, Seattle, Washington, United States of America; 2 AI for Good Lab, Microsoft, Redmond, Washington, United States of America; 3 Seattle Children’s Research Institute, Center for Integrative Brain Research, Seattle, Washington, United States of America; 4 Department of Paediatrics, Child and Youth Health, The University of Auckland, Auckland, New Zealand; Kobe University Graduate School of Medicine School of Medicine, JAPAN

## Abstract

**Background:**

Infection is thought to play a part in some infant deaths. Maternal infection in pregnancy has focused on chlamydia with some reports suggesting an association with sudden unexpected infant death (SUID).

**Objectives:**

We hypothesized that maternal infections in pregnancy are associated with subsequent SUID in their offspring.

**Setting:**

All births in the United States, 2011–2015

**Data source:**

Centers for Disease Control and Prevention (CDC) Birth Cohort Linked Birth-Infant Death Data Files.

**Study design:**

Cohort study, although the data were analysed as a case control study. Cases were infants that died from SUID. Controls were randomly sampled infants that survived their first year of life; approximately 10 controls per SUID case.

**Exposures:**

Chlamydia, gonorrhea and hepatitis C.

**Results:**

There were 19,849,690 live births in the U.S. for the period 2011–2015. There were 37,143 infant deaths of which 17,398 were classified as SUID cases (a rate of 0.86/1000 live births). The proportion of the control mothers with chlamydia was 1.7%, gonorrhea 0.2% and hepatitis C was 0.3%. Chlamydia was present in 3.8% of mothers whose infants subsequently died of SUID compared with 1.7% of controls (unadjusted OR = 2.35, 95% CI = 2.15, 2.56; adjusted OR = 1.08, 95% CI = 0.98, 1.19). Gonorrhea was present in 0.7% of mothers of SUID cases compared with 0.2% of mothers of controls (OR = 3.09, (2.50, 3.79); aOR = 1.20(0.95, 1.49)) and hepatitis C was present in 1.3% of mothers of SUID cases compared with 0.3% of mothers of controls (OR = 4.69 (3.97, 5.52): aOR = 1.80 (1.50, 2.15)).

**Conclusions:**

The marked attenuation of SUID risk after adjustment for a wide variety of socioeconomic and demographic factors suggests the small increase in the risk of SUID of the offspring of mothers with infection with hepatitis C in pregnancy is due to residual confounding.

## Introduction

In the United States (U.S.), sudden unexpected infant death (SUID) is a term that encompasses three separate causes of infant death as defined in the *International Classification of Diseases*, *10*^*th*^ *Revision* (ICD-10): sudden infant death syndrome (SIDS; R95), deaths from other ill-defined or unknown causes (R99), and accidental suffocation and strangulation in bed (W75). Approximately 3,500 infant deaths are classified as SUID cases annually in the U.S. [1].

As early as the 1960’s, researchers have suspected a role of infections in sudden infant death [2]. This association is consistent with the seasonality of SUID, with a greater prevalence in the winter [3], and the fact that the age of death peaks around 3 months, when infants are losing maternal immunity and their own immune system is immature [4].

Studies that have specifically investigated a link between sudden infant death and sexually transmitted infections have overwhelmingly focused on chlamydia. *Chlamydia trachomatis* is the most common bacterial pathogen of sexually transmitted infections in women in the U.S. with a prevalence in pregnant women ranging from 2–20% depending on the risk factors for the population in a given study [5]. Transmission of the infection can pass to the newborn from an infected cervix during birth. A published abstract from 1981 found significantly higher seropositivity for Chlamydia trachomatis in SIDS infants compared to controls [6]. Maternal seropositivity is associated with stillbirths, preterm delivery, premature rupture of the membranes, and low birthweight [7–10]. Another study found chlamydia inclusions in lung sections in 19% of SIDS cases compared with 3% of infants with a known cause of death [11].

The relationship between SUID and maternal sexually transmitted diseases and other infections is likely understudied because, individually, each is a rare occurrence, so it takes a very large dataset to have the necessary statistical power. Here we used the Centers for Disease Control and Prevention (CDC) Birth Cohort Linked Birth/Infant Death Data Set that includes every birth and death in the United States to study the association between SUID and various maternal infections in pregnancy, including chlamydia, gonorrhea and hepatitis C. We hypothesized that these maternal infections in pregnancy are associated with subsequent SUID in their offspring.

## Methods

### Study design

The study design is a population-based cohort study of all births and their mothers occurring in the U.S. from 2011 to 2015. Due to the very large sample size, infants that survived their first year of life (referred to as controls) were randomly sampled so there were approximately 10 controls for each SUID. The data were analysed as a case control study. Reporting of this study follows the STROBE (Strengthening the Reporting of Observational Studies in Epidemiology) guidelines [12].

### Data source

The cohort data were sourced from the publicly available Centers for Disease Control and Prevention (CDC) Birth Cohort Linked Birth-Infant Death Data Files [13]. Each contained several hundreds of features detailing various aspects of the mother’s and father’s background, the pregnancy and the birth of the infant. Additionally, the data includes details concerning the death of any infant in the first year of life.

### Exposure variable

The following maternal infections in pregnancy were examined: chlamydia, hepatitis C, hepatitis B, gonorrhea and syphilis. These are reported as Yes, No, Unknown or not stated.

### Outcome–SUID

Infants who died under 1 year of age and whose cause of death were certified as due to any one of the following International Classification of Diseases, 10th Revision (ICD10) codes:

R95 (sudden infant death syndrome, SIDS), R99 (other ill-defined and unspecified cause of mortality) or W75 (accidental suffocation and strangulation in bed). Deaths that occurred in infant’s first 7 days of life were excluded as the epidemiology is different from those dying between 7 and 365 days of life [14].

### Covariates

The analysis adjusted for the following variables, which are known to be associated with SUDI [15,16] and were in the linked dataset: mother’s age, mother’s education, mother’s race/Hispanic origin, marital status, smoking before pregnancy, smoking during pregnancy, delivery method, gestational age, sex of infant, father’s age, father’s race/Hispanic origin, live birth order, month prenatal care began and birthweight. The definition, origin and categorisation of these variables were:

- Mother’s Age [MAGER9]: <15, 15–19, 20–24, 25–29, 30–34, 35–39, 40–44, 45–49, 50–54 years
- Mother’s Education [MEDUC]: 8th grade or less, 9th through 12th grade with no diploma, high school graduate or GED completed, Some college credit, but not a degree, associate degree, bachelor’s degree, master’s degree, doctorate or professional degree, unknown
- Mother’s Race/Hispanic Origin [MRACEHISP]: Non-Hispanic White, Non-Hispanic Black, Non-Hispanic AIAN, Non-Hispanic Asian, Non-Hispanic NHOPI, Non-Hispanic more than one race, Hispanic, unknown or not stated

- Marital Status [DMAR]: Married, unmarried, unknown
- Smoking Before Pregnancy [CIG_0]: Number of cigarettes daily, categorized Yes, No, unknown or not stated
- Smoking During Pregnancy [CIG_1 (1^st^ trimester) or CIG_2 (2^nd^ trimester) or CIG_3 (3^rd^ trimester)]: Number of cigarettes daily, categorized Yes, No, unknown or not stated
- Delivery Method [DMETH_REC]: Vaginal, C-section, unknown
- Gestational age [GESTREC10]: <20 (observation deleted), 20–27, 28–31, 32–33, 34–36, 37–38, 39, 40, 41, 42 and over, unknown
- Sex of Infant [SEX]: Male, Female
- Admission to the neonatal intensive care unit [AB_NICU]: Yes, No, unknown or not stated
- Father’s Age [FAGE11]: <15, 15–19, 20–24, 25–29, 30–34, 35–39, 40–44, 45–49, 50–54, 55+ years, not stated
- Father’s Race/Hispanic Origin [FRACEHISP]: Non-Hispanic White, Non-Hispanic Black, Non-Hispanic AIAN, Non-Hispanic Asian, Non-Hispanic NHOPI, Non-Hispanic more than one race, Hispanic, unknown or not stated
- Live birth order [LBO_REC]: 1–7, 8+, unknown or not stated
- Month prenatal care began [PRECARE5]: 1st to 3rd month, 4th to 6th month, 7th to final month, No prenatal care, unknown or not stated
- Birth weight [BWTR14]: birthweight (g) 227–499, 500–749, 750–999, 1000–1249, 05 1250–1499, 1500–1999, 2000–2499, 2500–2999, 3000–3499, 3500–3999, 4000–4499, 4500–4999, 5000+, not stated

### Statistical analysis

Relative risks were estimated by calculation of odds ratios (OR). The univariable and multivariable ORs were obtained from unconditional logistic regression modelling as were their confidence intervals (CI). Population attributable risks (PAR) were calculated to estimate the unadjusted proportion of deaths explained by exposure to maternal infections [17].

## Results

**Fig 1:**
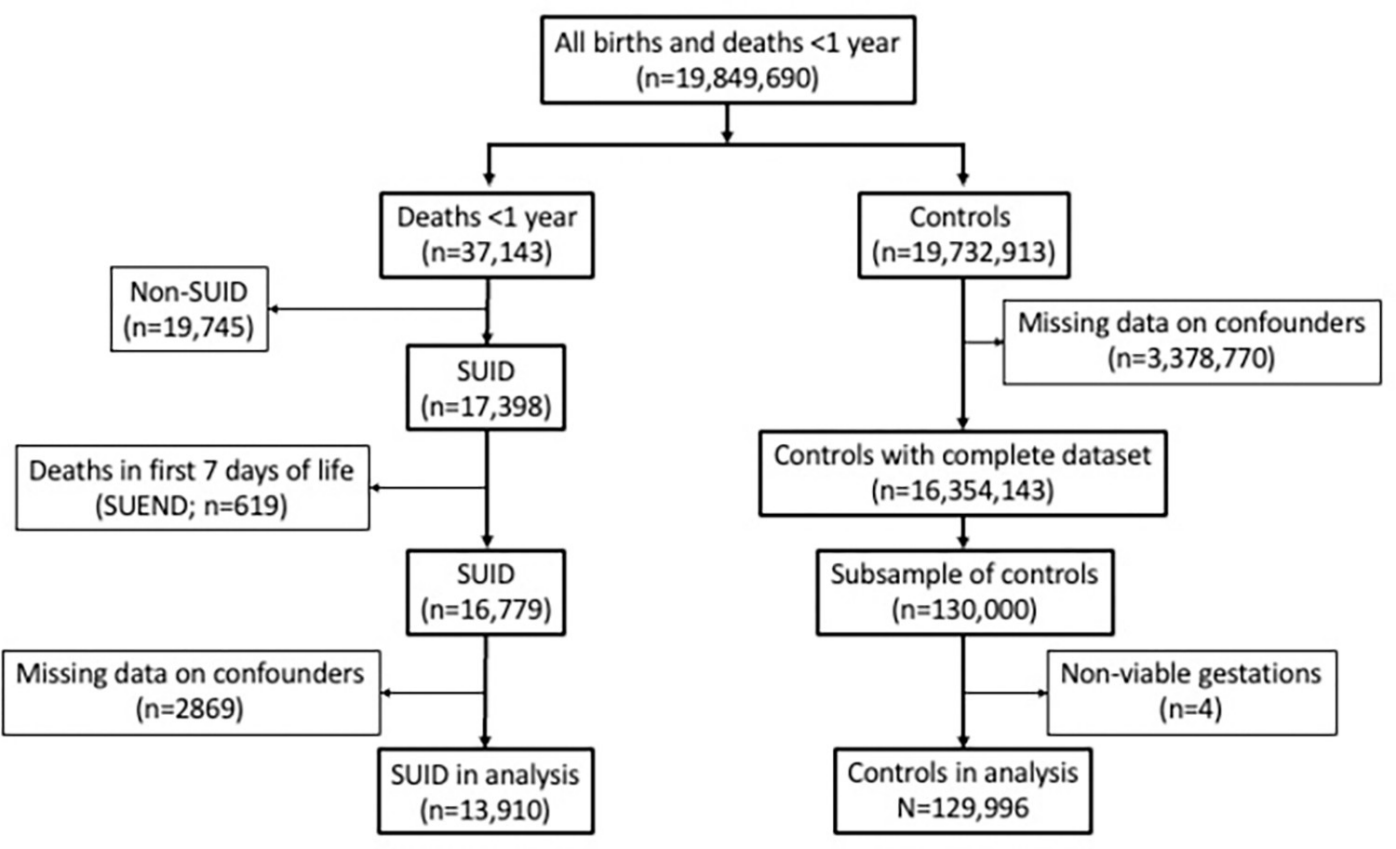
Flow diagram of SUDI cases and controls (infants that lived through to the first birthday and reasons for exclusions.

There were 19,849,710 live births in the U.S. for the period 2011–2015. The flow diagram of the cases and controls (infants that lived through to the first birthday) is shown in Fig 1.

### Cases

There were 37,143 infant deaths of which 17,398 were classified as SUID cases (a rate of 0.88/1000 live births). Of the SUID cases 619 occurred in the first week of life. Thus 16,779 SUID cases (96.4% of all SUID cases) were included in this analysis.

### Controls

Of the 19,732,933 infants born in the study period that survived to their first birthday a sample of 166,985 infants were randomly selected so that there was approximately 10 controls for each SUID case) This sample was used in this analysis.

Table 1 describes the characteristics of the study population (cases and controls).

**Table 1:** The study population: 16,779 SUID cases and 166,985 controls, which were randomly sampled so that there were approximately 10 controls for each SUID case). Controls were infants that survived to first birthday.

	Cases (N = 16779)		Controls (N = 166985)	
	N	%	N	%
**Mother’s Age**				
<15	32	0.2	109	0.1
15–19	2,205	13.1	11,628	7.0
20–24	6,236	37.2	37,503	22.5
25–29	4,644	27.7	47,758	28.6
30–34	2,538	15.1	44,065	26.4
35–39	903	5.4	20,850	12.5
40–45	210	1.3	4,732	2.8
45–49	10	0.1	319	0.2
50–54	1	0.0	21	0.0
**Mother’s Education**				
8th grade or less	412	2.5	5,951	3.6
9th through 12th grade with no diploma	3,696	22.0	18,203	10.9
High school graduate or GED completed	5,566	33.2	37,933	22.7
Some college credit, but not a degree	3,572	21.3	32,152	19.3
Associate degree	762	4.5	12,263	7.3
Bachelor’s degree	851	5.1	28,933	17.3
Master’s degree	279	1.7	12,729	7.6
Doctorate or Professional Degree	71	0.4	3,547	2.1
Unknown	1,570	9.4	15,274	9.1
**Mother’s Race/Hispanic Origin**				
Non-Hispanic White	7,742	46.1	82,374	49.3
Non-Hispanic Black	4,417	26.3	21,952	13.1
Non-Hispanic AIAN	300	1.8	5,764	3.5
Non-Hispanic Asian	281	1.7	4,631	2.8
Non-Hispanic NHOPI	60	0.4	364	0.2
Non-Hispanic more than one race	264	1.6	1,682	1.0
Hispanic	2,209	13.2	37,845	22.7
Origin unknown or not stated	1,506	9.0	12,373	7.4
**Marital Status**				
Married	5,220	31.1	99,786	59.8
Unmarried	11,559	68.9	67,199	40.2
**Smoking Before Pregnancy**				
No	9,528	56.8	131,001	78.5
Yes	6,472	38.6	29,864	17.9
Unknown	779	4.6	6,120	3.7
**Smoking During Pregnancy**				
No	10,046	59.9	134,730	80.7
Yes	5,950	35.5	26,123	15.6
Unknown	783	4.7	6,132	3.7
**Delivery Method**				
Vaginal	10,907	65.0	112,527	67.4
Cesarean	5,852	34.9	54,270	32.5
Unknown	20	0.1	188	0.1
**Gestational age**				
<27 weeks	243	1.4	797	0.5
28–31 weeks	571	3.4	1,893	1.1
32–33 weeks	578	3.4	2,462	1.5
34–36 weeks	2,417	14.4	13,446	8.1
37–38 weeks	4,464	26.6	41,663	25.0
39 weeks	4,095	24.4	50,065	30.0
40 weeks	2,380	14.2	33,059	19.8
41 weeks	1,041	6.2	14,217	8.5
42 weeks and over	958	5.7	9,228	5.5
Unknown	32	0.2	155	0.1
**Sex of Infant**				
Male	9,844	58.7	85,543	51.2
Female	6,935	41.3	81,442	48.8
**Admission to NICU**				
No	12,898	76.9	140,866	84.4
Yes	2,411	14.4	12,129	7.3
Unknown	1,470	8.8	13,990	8.4
**Father’s Age**				
<15	2	0.0	10	0.0
15–19	682	4.1	3,955	2.4
20–24	3,219	19.2	21,350	12.8
25–29	3,320	19.8	36,534	21.9
30–34	2,414	14.4	42,153	25.2
35–39	1,172	7.0	25,791	15.4
40–45	529	3.2	10,906	6.5
45–49	218	1.3	3,674	2.2
50–54	88	0.5	1,177	0.7
55+	44	0.3	485	0.3
Unknown	5,091	30.3	20,950	12.5
**Father’s Race/Hispanic Origin**				
Non-Hispanic White	5,382	32.1	72,741	43.6
Non-Hispanic Black	2,776	16.5	16,862	10.1
Non-Hispanic AIAN	204	1.2	4,919	2.9
Non-Hispanic Asian	186	1.1	3,820	2.3
Non-Hispanic NHOPI	44	0.3	322	0.2
Non-Hispanic more than one race	286	1.7	2,734	1.6
Hispanic	1,748	10.4	33,030	19.8
Origin unknown or not stated	5,971	35.6	30,411	18.2
Race unknown or not stated (Non-Hispanic)	182	1.1	2,146	1.3
**Live Birth Order**				
1	4,565	27.2	65,450	39.2
2	5,194	31.0	52,895	31.7
3	3,524	21.0	27,736	16.6
4	1,753	10.4	11,752	7.0
5	833	5.0	4,636	2.8
6	410	2.4	1,876	1.1
7	180	1.1	848	0.5
8+	204	1.2	881	0.5
Unknown	116	0.7	911	0.5
**Month Prenatal Care Began**				
1st to 3rd month	8,596	51.2	111,485	66.8
4th to 6th month	4,168	24.8	27,615	16.5
7th to final month	1,182	7.0	6,543	3.9
No prenatal care	606	3.6	2,172	1.3
Unknown or not stated	2,227	13.3	19,170	11.5
**Birth Weight (g)**				
<500	5	0.0	30	0.0
500–749	82	0.5	279	0.2
750–999	132	0.8	394	0.2
1000–1249	166	1.0	496	0.3
1250–1499	226	1.3	668	0.4
1500–1999	792	4.7	2,647	1.6
2000–2499	1,919	11.4	8,472	5.1
2500–2999	4,252	25.3	30,486	18.3
3000–3499	5,671	33.8	64,974	38.9
3500–3999	2,794	16.7	44,976	26.9
4000–4499	617	3.7	11,691	7.0
4500–4999	96	0.6	1,644	1.0
5000+	18	0.1	189	0.1
Not Stated	9	0.1	39	0.0

The proportion of the controls with chlamydia was 1.7%, gonorrhea 0.2% and hepatitis C was 0.3%. Due to small numbers, syphilis and hepatitis B were not considered further. Table 2 shows the characteristics of the exposures (chlamydia, gonorrhea, and hepatitis C).

**Table 2:** Characteristics of the exposures: Chlamydia (N = 3403), Gonorrhea (N = 502) and Hepatitis C (N = 663).

	Chlamydia (N = 3403)		Gonorrhoea (N = 502)		Hepatitis C (N = 663)	
**Mother’s Age**	**N**	**%**	**N**	**%**	**N**	**%**
<15	10	0.3	2	0.4	0	0.0
15–19	797	23.4	120	23.9	17	2.6
20–24	1587	46.6	215	42.8	163	24.6
25–29	677	19.9	106	21.1	250	37.7
30–34	259	7.6	49	9.8	163	24.6
35–39	64	1.9	9	1.8	56	8.4
40–45	9	0.3	1	0.2	14	2.1
45–49	0	0.0	0	0.0	0	0.0
50–54	0	0.0	0	0.0	0	0.0
**Mother’s Education**						
8th grade or less	119	3.5	12	2.4	21	3.2
9th through 12th grade with no diploma	934	27.4	161	32.1	146	22.0
High school graduate or GED completed	1353	39.8	196	39.0	268	40.4
Some college credit, but not a degree	711	20.9	97	19.3	159	24.0
Associate degree	126	3.7	17	3.4	29	4.4
Bachelor’s degree	105	3.1	14	2.8	21	3.2
Master’s degree	20	0.6	1	0.2	5	0.8
Doctorate or Professional Degree	3	0.1	0	0.0	0	0.0
Unknown	32	0.9	4	0.8	14	2.1
**Mother’s Race/Hispanic Origin**						
Non-Hispanic White	1142	33.6	116	23.1	515	77.7
Non-Hispanic Black	1261	37.1	280	55.8	43	6.5
Non-Hispanic AIAN	55	1.6	9	1.8	12	1.8
Non-Hispanic Asian	62	1.8	5	1.0	14	2.1
Non-Hispanic NHOPI	16	0.5	0	0.0	0	0.0
Non-Hispanic more than one race	60	1.8	13	2.6	10	1.5
Hispanic	733	21.5	66	13.1	61	9.2
Origin unknown or not stated	74	2.2	13	2.6	8	1.2
**Marital Status**						
Married	534	15.7	62	12.4	151	22.8
Unmarried	2869	84.3	440	87.6	512	77.2
**Smoking Before Pregnancy**						
No	2449	72.0	341	67.9	241	36.3
Yes	822	24.2	142	28.3	395	59.6
Unknown	132	3.9	19	3.8	27	4.1
**Smoking During Pregnancy**						
No	2580	75.8	354	70.5	248	37.4
Yes	694	20.4	127	25.3	388	58.5
Unknown	129	3.8	21	4.2	27	4.1
**Delivery Method**						
Vaginal	2470	72.6	340	67.7	438	66.1
Cesarean	933	27.4	162	32.3	225	33.9
Unknown	0	0.0	0	0.0	0	0.0
**Gestational age**						
<27 weeks	23	1	3	1	5	1
28–31 weeks	70	2.1	14	2.8	13	2.0
32–33 weeks	69	2.0	8	1.6	19	2.9
34–36 weeks	353	10.4	60	12.0	86	13.0
37–38 weeks	877	25.8	133	26.5	178	26.8
39 weeks	875	25.7	115	22.9	171	25.8
40 weeks	643	18.9	94	18.7	87	13.1
41 weeks	274	8.1	44	8.8	49	7.4
42 weeks and over	218	6.4	31	6.2	54	8.1
Unknown	1	0.0	0	0.0	1	0.2
**Sex of Infant**						
Male	1754	51.5	250	49.8	368	55.5
Female	1649	48.5	252	50.2	295	44.5
**Admission to NICU**						
No	3043	89.4	434	86.5	522	78.7
Yes	356	10.5	68	13.5	141	21.3
Unknown	4	0.1	0	0.0	0	0.0
**Father’s Age**						
<15	0	0.0	0	0.0	0	0.0
15–19	210	6.2	26	5.2	7	1.1
20–24	820	24.1	96	19.1	66	10.0
25–29	575	16.9	69	13.7	124	18.7
30–34	258	7.6	38	7.6	106	16.0
35–39	111	3.3	20	4.0	68	10.3
40–45	42	1.2	8	1.6	36	5.4
45–49	11	0.3	4	0.8	20	3.0
50–54	2	0.1	1	0.2	6	0.9
55–98	2	0.1	1	0.2	6	0.9
Unknown	1372	40.3	239	47.6	224	33.8
**Father’s Race/Hispanic Origin**						
Non-Hispanic White	636	18.7	56	11.2	294	44.3
Non-Hispanic Black	670	19.7	149	29.7	38	5.7
Non-Hispanic AIAN	32	0.9	5	1.0	8	1.2
Non-Hispanic Asian	35	1.0	5	1.0	8	1.2
Non-Hispanic NHOPI	11	0.3	0	0.0	2	0.3
Non-Hispanic more than one race	51	1.5	5	1.0	7	1.1
Hispanic	594	17.5	39	7.8	58	8.7
Origin unknown or not stated	1357	39.9	241	48.0	242	36.5
Race unknown or not stated (Non-Hispanic)	17	0.5	2	0.4	6	0.9
**Live Birth Order**						
1	1534	45.1	204	40.6	184	27.8
2	977	28.7	142	28.3	176	26.5
3	500	14.7	96	19.1	138	20.8
4	221	6.5	35	7.0	87	13.1
5	85	2.5	11	2.2	43	6.5
6	36	1.1	6	1.2	16	2.4
7	18	0.5	3	0.6	7	1.1
8+	14	0.4	2	0.4	10	1.5
Unknown	18	0.5	3	0.6	2	0.3
**Month Prenatal Care Began**						
1st to 3rd month	1888	55.5	269	53.6	339	51.1
4th to 6th month	1067	31.4	160	31.9	176	26.5
7th to final month	277	8.1	43	8.6	85	12.8
No prenatal care	63	1.9	20	4.0	27	4.1
Unknown or not stated	108	3.2	10	2.0	36	5.4
**Birth Weight (g)**						
<500	0	0.0	0	0.0	0	0.0
500–749	7	0.2	0	0.0	2	0.3
750–999	14	0.4	3	0.6	1	0.2
1000–1249	11	0.3	2	0.4	3	0.5
1250–1499	22	0.6	3	0.6	3	0.5
1500–1999	100	2.9	21	4.2	37	5.6
2000–2499	267	7.8	48	9.6	96	14.5
2500–2999	785	23.1	122	24.3	198	29.9
3000–3499	1355	39.8	191	38.0	211	31.8
3500–3999	673	19.8	97	19.3	98	14.8
4000–4499	159	4.7	15	3.0	12	1.8
4500–4999	8	0.2	0	0.0	2	0.3
5000+	2	0.1	0	0.0	0	0.0
Not Stated	0	0.0	0	0.0	0	0.0

Chlamydia was present in 3.8% of mothers whose infants subsequently died of SUID compared with 1.7% of mothers whose infants who did not die (unadjusted OR = 2.35, 95% CI = 2.15, 2.56 and adjusted OR = 1.08, 95% CI = 0.98, 1.19; Table 3).

**Table 3:** Number, percentage and unadjusted and adjusted odds ratios (OR) and their 95% confidence intervals (CI) for chlamydia, gonorrhea and hepatitis C.

	Case		Control		Unadjusted		Adjusted *	
	N	%	N	%	OR	(95% CI)	OR	(95% CI)
**Chlamydia**								
Yes	634	3.8	2,769	1.7	2.35	(2.15, 2.56)	1.08	(0.98, 1.19)
No	14,643	87.3	150,046	89.9	Ref	Ref	Ref	Ref
Unknown	1,502	9.0	14170	8.5	1.09	(1.03, 1.15)	0.73	(0.56, 0.93)
**Gonorrhea**								
Yes	118	0.7	384	0.2	3.09	(2.50, 3.79)	1.20	(0.95, 1.49)
No	15,159	90.3	152,431	91.3	Ref	Ref	Ref	Ref
Unknown	1,502	9.0	14170	8.5	1.07	(1.01, 1.13)	0.73	(0.56, 0.93)
**Hepatitis C**								
Yes	210	1.3	453	0.3	4.69	(3.97, 5.52)	1.80	(1.50, 2.15)
No	15,067	89.8	152362	91.2	Ref	Ref	Ref	Ref
Unknown	1,502	9.0	14170	8.5	1.07	(1.01, 1.13)	0.73	(0.57, 0.94)

* Adjusted for mother’s age, mother’s education, mother’s race/hispanic origin, marital status, smoking before pregnancy, smoking during pregnancy, delivery method, gestational age, sex of infant, admission to neonatal intensive care unit, month prenatal care began, father’s age, father’s race/Hispanic origin, birth weight, and live birth order.

Gonorrhea was present in 0.7% of mothers of SUID cases compared with 0.2% of mothers of controls (unadjusted OR = 3.09, 95% CI = 2.50, 3.79 and adjusted OR = 1.20, 95% CI = 0.95, 1.49) and hepatitis C was present in 1.3% of mothers of SUID cases compared with 0.3% of mothers of controls (unadjusted OR = 4.69, 95% CI = 3.97, 5.52 and adjusted OR = 1.80, 95% CI = 1.50, 2.15).

Table 4 shows the adjusted covariates for chlamydia. The covariates for gonorrhea and hepatitis C are similar (not shown).

**Table 4:** Adjusted covariates for chlamydia.

Factor	adjusted OR*	(95% CI)
**Mother’s Age**		
<15	5.44	(3.52, 8.20)
15–19	2.60	(2.39, 2.83)
20–24	2.00	(1.88, 2.13)
25–29	1.40	(1.33, 1.49)
30–34	Ref	
35–39	0.73	(0.68, 0.80)
40–45	0.64	(0.55, 0.75)
45–49	0.44	(0.22, 0.80)
50–54	0.78	(0.04, 3.92)
**Mother’s Education**		
8th grade or less	1.17	(1.02, 1.33)
9th through 12th grade with no diploma	1.81	(1.65, 1.97)
High school graduate or GED completed	1.64	(1.51, 1.78)
Some college credit, but not a degree	1.60	(1.47, 1.73)
Associate degree	1.31	1.18, 1.45
Bachelor’s degree	Ref	
Master’s degree	0.92	(0.80, 1.05)
Doctorate or Professional Degree	0.92	(0.71, 1.16)
Unknown	1.25	(1.04, 1.50)
**Smoking During Pregnancy**		
No	Ref	
Unknown	1.26	(0.84, 1.90)
Yes	1.67	(1.52, 1.84)
**Smoking Before Pregnancy**		
No	Ref	
Unknown	1.01	(0.67, 1.52)
Yes	1.51	(1.37, 1.65)
**Marital status**		
Not Married	Ref	
Married	1.49	1.43, 1.56
**Admitted to NICU**		
No	Ref	
Unknown	0.74	(0.56, 0.97)
Yes	1.16	(1.09, 1.24)
**Gestation**		
<27 weeks	1.14	(0.89, 1.44)
28–31 weeks	1.35	(1.17, 1.55)
32–33 weeks	1.23	(1.09, 1.39)
34–36 weeks	1.30	(1.21, 1.40)
37–38 weeks	1.14	(1.07, 1.20)
39 weeks	1.05	(0.99, 1.11)
40 weeks	Ref	
41 weeks	1.03	(0.95, 1.11)
42 weeks and over	1.12	(1.03, 1.21)
Unknown	1.17	(0.70, 1.87)
**Mother’s Race Hispanic Origin**		
Non-Hispanic White	Ref	
Non-Hispanic Black	1.06	(1.00, 1.12)
Non-Hispanic AIAN	1.00	(0.84, 1.18)
Non-Hispanic Asian	0.95	(0.81, 1.13)
Non-Hispanic NHOPI	1.26	(0.88, 1.80)
Non-Hispanic more than one race	1.16	(1.00, 1.34)
Hispanic	0.70	(0.65, 0.76)
Origin unknown or not stated	1.17	(1.05, 1.30)
**Sex**		
Male	Ref	
Female	1.42	(1.38, 1.47)
**Delivery Method**		
Cesarean	Ref	
Unknown	0.88	(0.51, 1.45)
Vaginal	0.90	(0.87, 0.94)
**Birth Weight (g)**		
<500	2.24	(0.74, 5.63)
500–749	2.33	(1.65, 3.28)
750–999	2.63	(1.99, 3.47)
1000–1249	2.37	(1.88, 2.98)
1250–1499	2.34	(1.92, 2.83)
1500–1999	2.16	(1.93, 2.41)
2000–2499	1.86	(1.73, 1.98)
2500–2999	1.32	(1.26, 1.38)
3000–3499	Ref	
3500–3999	0.81	(0.77, 0.85)
4000–4499	0.73	(0.67, 0.80)
4500–4999	0.85	(0.68, 1.04)
5000+	1.12	(0.65, 1.81)
Not Stated	3.39	(1.23, 8.93)
**Father’s Race Hispanic Origin**		
Non-Hispanic White	Ref	
Non-Hispanic Black	1.25	1.17, 1.34
Non-Hispanic AIAN	0.79	0.65, 0.97
Non-Hispanic Asian	0.91	0.74, 1.11
Non-Hispanic NHOPI	1.13	0.74, 1.69
Non-Hispanic more than one race	0.92	0.81, 1.06
Hispanic	0.65	0.59, 0.71
Origin unknown or not stated	0.92	0.86, 0.99
Race unknown or not stated (Non-Hispanic)	0.74	0.62, 0.87
**Father’s Age**		
<15	1.18	0.16, 5.58
15–19	1.48	1.33, 1.66
20–24	1.34	1.26, 1.44
25–29	1.13	1.07, 1.20
30–34	Ref	
35–39	0.92	0.85, 0.99
40–45	0.96	0.86, 1.06
45–49	1.02	0.87, 1.19
50–54	1.17	0.92, 1.48
55–98	1.34	0.95, 1.86
Unknown	1.68	1.55, 1.81
**Live Birth Order**		
1	Ref	
2	1.93	1.84, 2.02
3	2.57	2.43, 2.71
4	3.10	2.89, 3.32
5	3.67	3.34, 4.02
6	4.40	3.87, 4.99
7	4.73	3.93, 5.68
8	5.84	4.87, 6.96
Unknown	1.93	1.55, 2.39
**Month Prenatal Care Began**		
1st to 3rd month	Ref	
4th to 6th month	1.31	1.25, 1.36
7th to final month	1.45	1.35, 1.56
No prenatal care	1.51	1.36, 1.68
Unknown or not stated	1.27	1.16, 1.38

* Adjusted for all the variables in the table plus Chlamydia.

The population attributable fractions for chlamydia, gonorrhea and hepatitis C are 2.2%, 0.5% and 1.0% respectively.

## Discussion

This study found that maternal infection with chlamydia, gonorrhea and hepatis C during pregnancy was associated with an increased risk of SUID in the offspring. After adjustment for a wide range of sociodemographic and other risk factors for SUID, the magnitude of the risk was attenuated, but remained statistically significant for hepatitis C.

Some infections, for example syphilis and HIV (human immunodeficiency virus), can pass across the placenta and infect the fetus in utero. Other sexually transmitted diseases, like gonorrhea, chlamydia, hepatitis B, and genital herpes, can pass from the mother to the baby as the baby passes through the genital tract. Our analysis adjusted for mode of delivery. A meta-analysis found that cesarean delivery did not decrease the risk of perinatal transmission of hepatitis C virus from positive mothers [18]. One possible explanation is that vaginal birth does not simply increase the risk of transmitted infections, but it may also convey increased protection against infections, for example through the gut microbiome. There is increasing evidence that infants born by Cesarean delivery lack bacteria that are important for the stabilization of the gut microbiota [19]. Clearly, our study cannot provide insights into causal relationships. The most likely explanation for our findings is residual confounding.

This study has shown that these maternal infections in pregnancy are associated with young maternal age, less maternal education, unmarried, Hispanic and Black race, and maternal smoking both before and during pregnancy. All these factors are also known risk factors for SUID. We speculate that this may account for previous findings of a higher seropositivity for chlamydia^6^ and greater frequency of chlamydia inclusions in the lungs of SUID cases [11].

Limitations of this study must be considered. The prevalence of these infections is at the lower limits of that reported from other studies, which may reflect those other studies were conducted for more selected and higher risk populations. In contrast this study was unbiased as it considered the entire population of births in the U.S. over 5 years. The infections identified within our study may reflect not only identification of the infections but also that they have been treated. The dataset does not record information on whether they have been treated. A further limitation is the missing data for the covariates, but since the loss was similar in both cases and controls for both the exposures (9.0% and 8,5% respectively) and covariates, with the exception of father’s age and origin, it is unlikely to have affected the results. Father’s age was unknown for 30.3% of cases and 12.5% of controls and father’s origin was unknown for 35.6% and 18.4% respectively. This reflects that SUID is more common for parents that are unmarried and disadvantaged.

This study has examined chlamydia, gonorrhea and hepatitis C. There were insufficient numbers to examine syphilis and hepatitis B. Other infections, such as bacterial vaginosis and human immunodeficiency virus (HIV), are not reported in the publicly available CDC Birth Cohort Linked Birth-Infant Death Data Files. Bacterial vaginosis may be a cause of SUID as it causes preterm birth and low birthweight [20], which are established risk factors for SUID. This suggests maternal infection, especially relating to the genital tract in pregnancy should not be entirely discounted.

## Conclusions

We conclude that even if there is a causal link, the number of cases that could be attributed to these infections is small. This does not negate the fact that these infections in pregnancy are important and cause a wide range of health problems to the mother, fetus, and infant. In conclusion, even though we argue that SUID is not a direct consequence of these infections, paying a holistic assessment of the mother’s health conditions and being extra vigilant on an infant during early months of birth may be beneficial.

## Supporting information

S1 Dataset (ZIP)
